# ACUTE ABDOMEN systemic sonographic approach to acute abdomen in emergency department: a case series

**DOI:** 10.1186/s13089-019-0136-5

**Published:** 2019-09-23

**Authors:** Maryam Al Ali, Sarah Jabbour, Salma Alrajaby

**Affiliations:** 1 Arab Board in Emergency Medicine, Rashid Hospital Trauma Center, Dubai Health Authority, oud metha, Dubai, United Arab Emirates; 2 Emergency Medicine Resident, Rashid Hospital Trauma Center, Dubai Health Authority, oud metha, Dubai, United Arab Emirates

**Keywords:** Acute abdomen, POCUS, Emergency ultrasound, Critical ultrasound

## Abstract

**Background:**

Acute abdomen is a medical emergency with a wide spectrum of etiologies. Point-of-care ultrasound (POCUS) can help in early identification and management of the causes. The ACUTE–ABDOMEN protocol was created by the authors to aid in the evaluation of acute abdominal pain using a systematic sonographic approach, integrating the same core ultrasound techniques already in use—into one mnemonic. This mnemonic ACUTE means: A: abdominal aortic aneurysm; C: collapsed inferior vena cava; U: ulcer (perforated viscus); T: trauma (free fluid); E: ectopic pregnancy, followed by ABDOMEN which stands: A: appendicitis; B: biliary tract; D: distended bowel loop; O: obstructive uropathy; Men: testicular torsion/Women: ovarian torsion. The article discusses two cases of abdominal pain the diagnosis and management of which were directed and expedited as a result of using the ACUTE–ABDOMEN protocol. The first case was of a 33-year-old male, who presented with a 3-day history of abdominal pain, vomiting and constipation. Physical exam revealed a soft abdomen with generalized tenderness and normal bowel sounds. Laboratory tests were normal. A bedside ultrasound done using the ACUTE–ABDOMEN protocol showed signs of intussusception. This was confirmed by CT-abdomen. The second case was of a 70-year-old female, a known case of diabetes and hypertension, who presented with a 3-hour history of abdominal pain, vomiting and diarrhea. She had a normal physical exam and laboratory studies. Her symptoms mimicking simple gastroenteritis had improved. However, bedside ultrasound, using the ACUTE–ABDOMEN protocol showed localized free fluid with dilated small bowel loop in right lower quadrant with absent peristalsis. A CT abdomen confirmed a diagnosis of intestinal obstruction. These two cases demonstrate that the usefulness of applying POCUS in a systematic method—like the “ACUTE–ABDOMEN” approach—can aid in patient diagnosis and management.

**Case presentation:**

We are presenting two cases of undifferentiated acute abdomen pain, where ACUTE ABDOMEN sonographic approach was applied and facilitated the accurate patient management and disposition.

**Conclusion:**

ACUTE ABDOMEN sonographic approach in acute abdomen can play an important role in ruling out critical diagnosis, and can guide emergency physician or any critical care physician in patient management.

## Background

The use of bedside ultrasound by physicians has become increasingly popular in the last two decades, even more so in critically ill patients. In emergency medicine, point of care ultrasound (POCUS) is being used as a diagnostic modality, given its easy accessibility and non-invasive nature. It is used as an adjunct, directing physicians on further testing modalities and treatment plans.

However, there has always been the question of reliability of ultrasound being performed by non-radiologists. In a study involving 651 patients complaining of renal colic, emergency physicians were found to have moderate to high sensitivity for identifying hydronephrosis on POCUS when compared with the consensus interpretation of the same studies by emergency radiologists [1].

Emergency ultrasound is now considered a core skill for emergency physician. American College of Emergency Physicians has introduced Emergency Ultrasound Guidelines that were introduced in 2008 and revisited in 2016, acknowledging that emergency ultrasound is a part of patient assessment within different clinical categories.

Given the time constraint and critical conditions of patients in the emergency, ultrasound approach in the ED should be focused, systemic and specific to patient symptom. Thus, the different protocols for different patient symptom came into practice. These fall under ACEP’s functional clinical category of “symptom or sign-based ultrasound”. To mention a few, FAST ultrasound for the trauma patient, RUSH protocol for the hypotensive patient, and BLUE protocol for the dyspneic patient [2].

Similarly, the ACUTE ABDOMEN protocol [3] was created by the authors to aid in the evaluation of acute abdominal pain using a systematic sonographic approach, integrating the same core ultrasound techniques already in use, into one mnemonic. This novel approach to the patient with abdominal pain systematically assesses the five critical causes—in the first part of the mnemonic “ACUTE” followed by scanning for other surgical causes in the second part of the mnemonic “ABDOMEN”.

The mnemonic ACUTE stands for—A: abdominal aortic aneurysm, C: collapsed inferior vena cava, U: ulcer (perforated viscus), T: trauma (free fluid), E: ectopic pregnancy, followed by ABDOMEN which stands for—A: appendicitis, B: biliary tract, D: distended bowel loop, O: obstructive uropathy, Men: testicular torsion, Women: ovarian torsion.

This might seem quite overwhelming and time consuming for an already busy ER, but if done in the proposed systemic approach, it can, on the contrary, provide pertinent information in a short time. An example of how the ACUTE ABDOMEN protocol directed the patient management is demonstrated in the two cases discussed below.

## Case presentation

### Case 1

A 33-year-old male of Asian (Pakistani) origin was presented to Rashid Emergency and Trauma Center with a complaint of sudden non-radiating epigastric abdominal pain. The pain was associated with vomiting and constipation for 3 days. There was no chest pain, diarrhea, fever or melena.

The patient had similar presentation in his country 4 years ago and was managed conservatively.

Vital signs were normal. Abdomen was soft with generalized tenderness and normal bowel sounds.

Laboratory studies were normal.

**Fig. 1:**
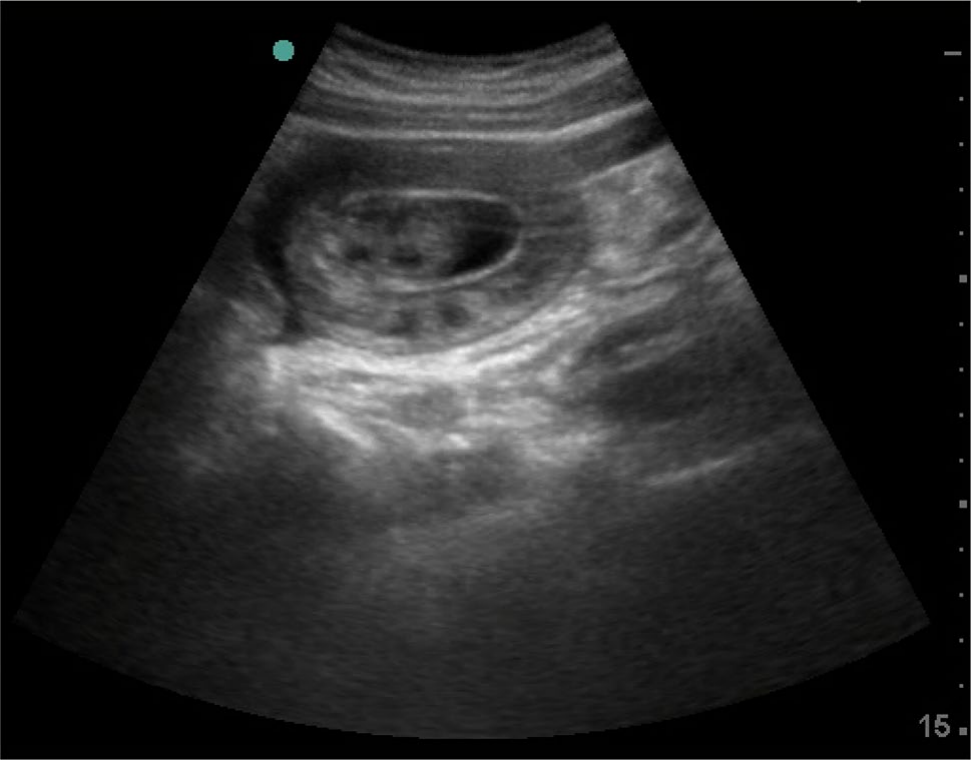
Point of care ultrasound for acute abdomen (ACUTE approach) showed localized free fluid with doughnut sign edematous bowel loop wall

**Fig. 2:**
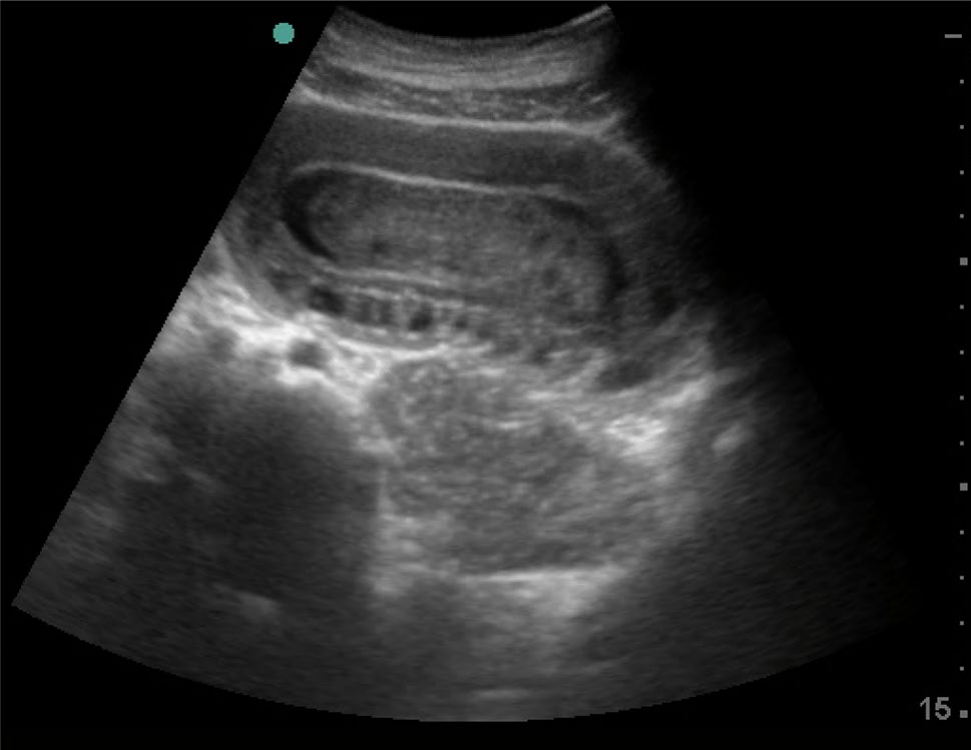
Point of care ultrasound for acute abdomen (ACUTE approach) showed bowel telescoping inside itself with edematous bowel loop wall (long axis)

Point of care ultrasound for acute abdomen (ACUTE approach) showed:

- A: normal aortic diameter
- C: collapsed IVC > 50%,
- U: no free air
- T: localized free fluid with doughnut sign (Fig. 1), and long axis of the segment showed bowel telescoping inside itself with oedematous bowel loop wall (Fig. 2).

#### Abdominal X-ray was normal

**Fig. 3:**
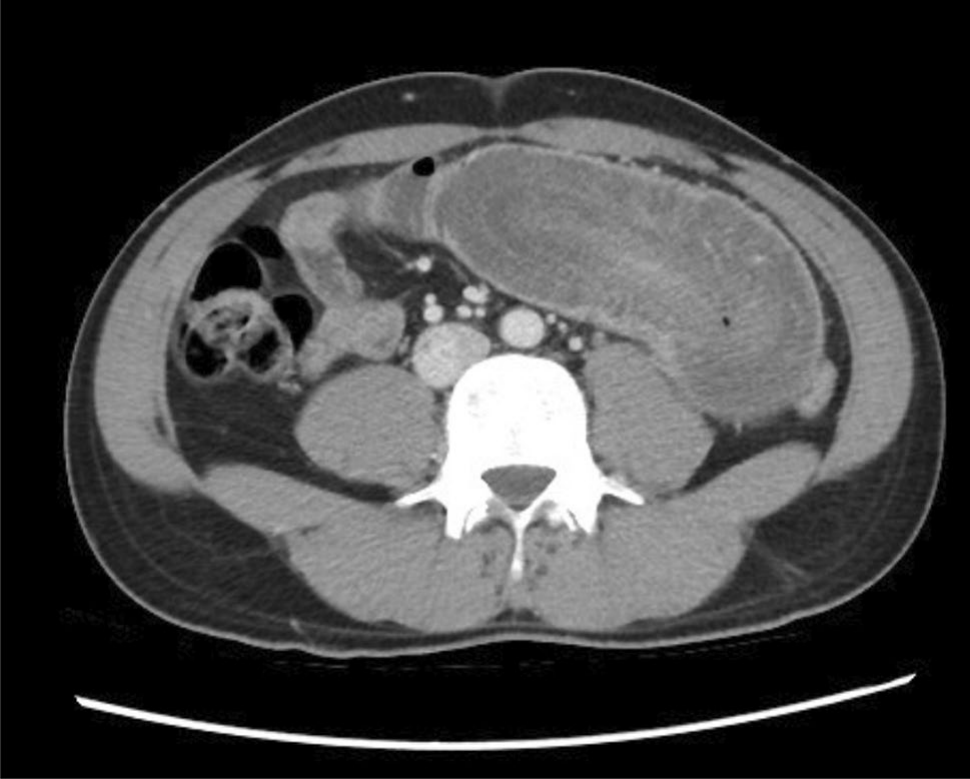
CT scan abdomen with contrast showed a long segment entero-enteric (jejuno-jejunal) intussusception seen mainly in the left side of the abdomen

After ultrasound finding, we decided to perform urgent CT scan of the abdomen which showed the evidence of long segment entero-enteric (jejuno-jejunal) intussusception seen mainly in the left side of the abdomen, with relatively collapsed intussusceptum segment. Mild amount of intraperitoneal free fluid was seen in the jejunal mesentery next to the involved segments as well as in the pelvic peritoneal pouches (Fig. 3).

Surgical consultation was obtained; however, the patient preferred to continue the medical care in his country.

### Case 2

A 70-year-old female diabetic and hypertensive was presented to Rashid Emergency and Trauma Center with generalized abdominal pain for 3 h after food which was associated with vomiting and diarrhea twice. There was no urinary complaint, chest pain or fever.

Vital signs were normal. Abdomen was soft with mild tenderness in the right lower abdomen, without peritonitis clinical sign. Laboratory test was insignificant, except for leukocytosis 17.2·10^3^/μL (3.6–11.0·10^3^/μL) and plasma lactic acid 3.7 mmol/L (0.5–2.2 mmol/L).

**Fig. 4:**
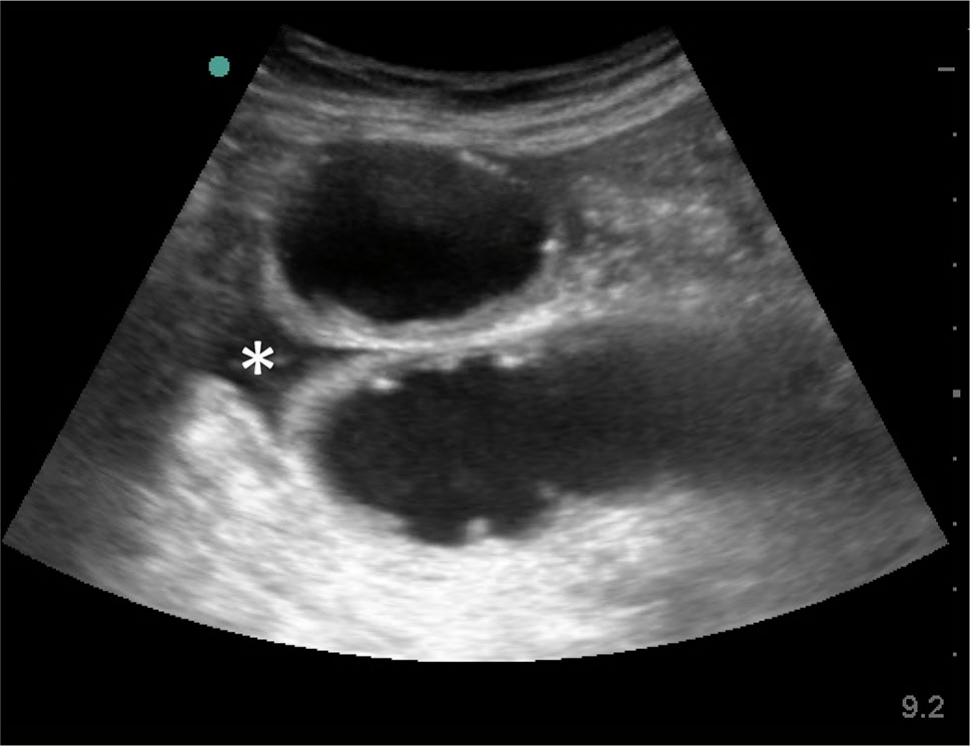
Point of care ultrasound for acute abdomen showed localized free fluid * with dilated small bowel loop in right lower quadrant with absent peristalsis

Point of care ultrasound for acute abdomen (ACUTE approach) showed:

- A: normal aortic diameter
- C: normal IVC
- U: no free air
- T: localized free fluid with dilated small bowel loop in right lower quadrant with absent peristalsis (Fig. 4).

**Fig. 5:**
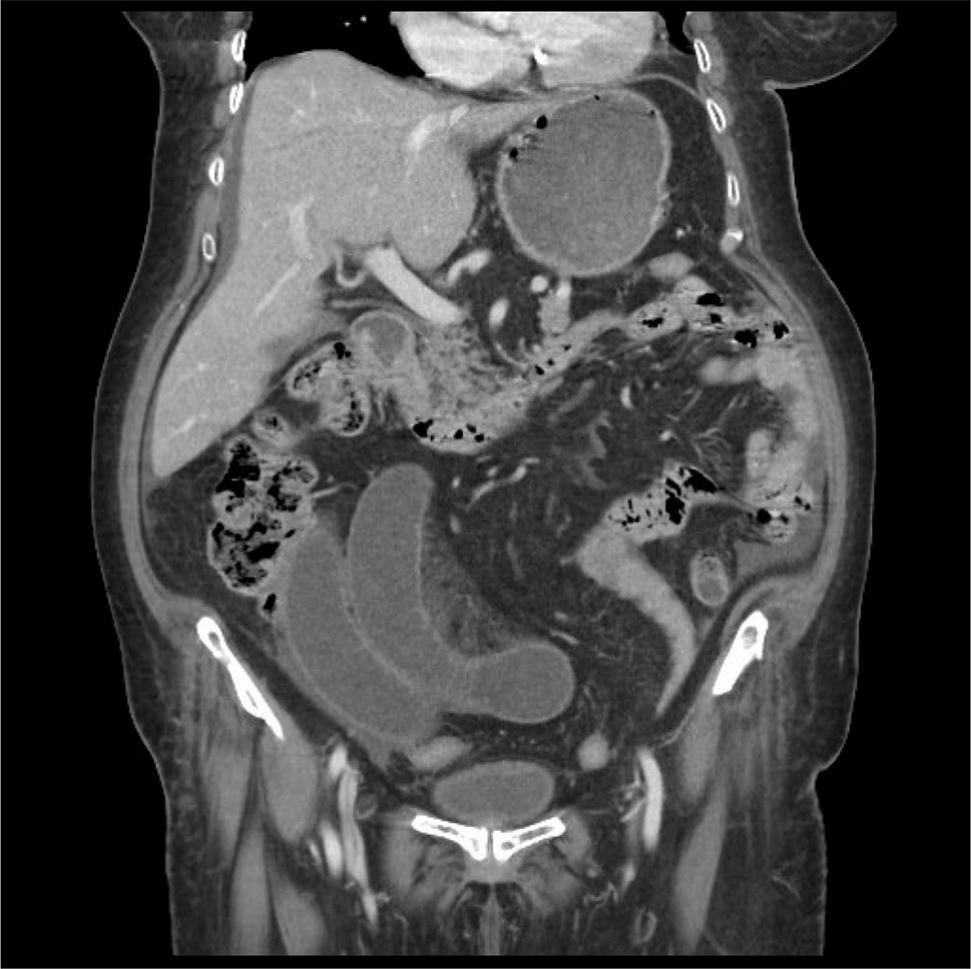
CT abdomen with contrast showed dilated small bowel loops in the right side of the lower abdomen and pelvis with a short segment critical stenosis measuring about 2.3 cm

CT abdomen showed evidence of significantly dilated small bowel loops in the right side of the lower abdomen and pelvis with a short segment critical stenosis measuring about 2.3 cm. It also showed an evidence suggestive of internal herniation with dilated loops of bowel. Jejunal loops and distal ileum appear collapsed. Free fluid was seen in the abdomen and pelvis (Fig. 5).

Urgent surgical consultation was obtained and the patient had undergone laparotomy which was conclusive of an omental band adherent to the small bowel mesentery.

About 150 cm proximal to ileo cecal juction producing congestion with punctuate haemorrhages and dilatation of the proximal bowel of about 30 cm. After the band was released; the color of the bowel changed to normal with good peristalsis. No bowel resection was needed.

Patient post-operative course was uneventful, and she was discharged home after 5 days.

Patient symptoms that mimicked simple gastroenteritis had improved, but because of our ultrasound finding that suggested intestinal obstruction, it was decided to perform a CT abdomen. If not for our preliminary ultrasound findings, this patient would have been discharged without further imaging.

## Discussion

POCUS approaches have been published in multiple critical care conditions such as Bedside Lung Ultrasound in Emergency (BLUE) protocol in respiratory failure which concluded that lung ultrasound immediately provided the diagnosis of acute respiratory failure in 90.5% of cases. The BLUE protocol focused on lung ultrasound only [4]; however, the RUSH protocol demonstrated that initial integration of bedside ultrasound into the evaluation of the patient with shock results in a more accurate initial diagnosis with an improved patient care plan [5].

In this case series, we presented two cases where ACUTE ABDOMEN played an important role in their management, POCUS can play an important role in acute abdomen, by early recognition and diagnosing critical conditions. Instead of relying only on physical examination which might have very low sensitivity in comparison to ultrasound, for example in physical examination can identify 38% of patients with AAA [6], ultrasound by emergency physician can detect AAA in 93% to 100% of cases, with specificities approaching 100% [7–9].

In other pathologies like pneumoperitoneum, X-ray is more frequently used, but ultrasound is superior to upright chest and lateral decubitus X-ray where the sensitivity is 92% versus 78% for X-ray [10].

The two cases presented with undifferentiated acute abdomen, in which bedside ultrasound was started with ACUTE approach after history and physical examination, (Table 1A) [11] showed unexpected finding, which needed further radiological evaluation by CT scan with contrast and early referral to surgical team.

**Table 1:** ACUTE ABDOMEN sonographic aproach: findings and techniques

	Pathology	Finding	Technique
A. ACUTE			
A	Abdominal aortic aneurysm	Abdominal aortic > 3 cm?	Probe: curvilinear or phased array Scan: long axis and short axis from epigastric till the bifurcation of common iliac
C	Collapsed inferior vena cave (assessment of patient’s volume status)	IVC collapsing > 50%?	Probe: curvilinear or phased array Scan: subxiphoid long axis, assessing the respiratory dynamics of the IVC
U	Ulcer (perforated viscus)	Pneumoperitoneum? Direct sign: Increased echogenicity of peritoneal stripe Presence of A lines Indirect sign: Intraperitoneal free fluid Air bubbles in ascetic fluid Thickened bowel loop Bowel or gallbladder thickened wall with ileus	Probe: curvilinear or high frequency linear Scan: epigastrium through the right upper quadrant (RUQ) along the transverse and longitudinal axes
T	Trauma (free fluid)	Intraperitoneal hypoechoic fluid?	Probe: curvilinear or phased array Scan: right upper quadrant, left upper quadrant, suprapubic Localized free fluid: scan right and left paracolic gutter
E	Ectopic pregnancy (empty uterus)	Intraperitoneal hypoechoic fluid, empty uterus or extra-uterine gestational sac?	Probe: curvilinear Scan suprapubic long and short axis
B. ABDOMEN			
A	Appendicitis	Non compressible Diameter > 6 mm	Probe: high frequency linear Scan: right lower abdomen
B	Biliary tract	Cholecystitis: Precystic fluid Sonographic murphy Gallbladder calculi Choledocholithiasis CBD > 6 mm	Probe: curvilinear or phased array Scan: right upper abdomen
D	Distended bowel loop	Small bowel obstruction? Dilated small bowel loop > 3 cm Back-and-forth movement of spot echoes inside fluid-filled bowel Decrease bowel peristalsis	Probe: curvilinear or high frequency linear Scan: epigastrium, bilateral colic gutters, and suprapubic regions
O	Obstructive uropathy	Hydronephrosis? Dilated renal calyces Renal stone: acoustic echogenic foci urterovesical junction.	Probe: curvilinear Scan: Longitudinal view Lower intercostal, right: mid axillary line, left: posterior axillary line.
MEN	Men: testicular torsion	Hypoechoic testis compare to normal Reduce or no perfusion	Probe: high frequency linear Scan: scrotal transverse and longitudinal Doppler
	Women: ovarian torsion	Adnexal mass > 4 cm Pelvic free fluid Reduced blood flow on Doppler	Probe: curvilinear Scan: suprapubic, sagittal and transverse identify uterus, then move right and left

For the second part of the approach, ABDOMEN (Table 1B) [11] usually will be a secondary survey and will focus the assessment with guide of the history and examination.

## Conclusions

ACUTE and ABDOMEN sonographic approach in acute abdomen can play an important role in ruling out critical diagnosis, and can guide emergency physician or any critical care physician in patient management.

Further prospective studies are warranted.

## Data Availability

Not applicable.

## Abbreviations

POCUS: point of care ultrasound
BLUE: bedside lung ultrasound in emergency
RUSH: rapid ultrasound in shock
AAA: abdominal aortic aneurysm
IVC: inferior vena cava
